# Current status of nonsuicidal injuries and associated factors among junior high school students in Hainan Province, China: a cross-sectional study

**DOI:** 10.1186/s40359-023-01227-x

**Published:** 2023-07-05

**Authors:** Siying Ma, Zhaoxia Su

**Affiliations:** 1 Department of Hospital Infection-control, the First Hospital of Hainan Medical University, Haikou City, Hainan Province 570102 China; 2 Department of Psychology, the First Hospital of Hainan Medical University, No. 29 Yilong West Road, Longhua District, Haikou City, Hainan Province 570102 China

**Keywords:** Nonsuicidal self-injury, Adolescent, Junior high school students

## Abstract

**Background:**

To summarize the general status of nonsuicidal self-injury (NSSI) behaviour and the characteristics of junior high school students and to determine the risk factors associated with NSSI behaviour.

**Methods:**

Five middle schools in the rural and urban areas of Hainan Province were randomly selected for this cross-sectional study, and junior high school students were administered questionnaires, including the General Sociodemographic Questionnaire, Ottawa Self-Injury Scale, Anxiety Self-Rating Scale, Depression Self-Rating Scale, Adolescent Lifestyle Scale, and Parenting Style Scale.

**Results:**

The NSSI rate among junior high school students in Hainan Province was 28.9%, with a higher prevalence among girls than boys (P < 0.05). The age range was 11–16 years, with a mean age of 13.08 ± 0.911 years. The most common form of self-injury was scratching/bruising, followed by hitting oneself, pulling out hair, biting, head banging, and cutting. The NSSI methods of scratching/bruising, hitting oneself and cutting more commonly occurred in girls than boys (P < 0.05). The most common sites of self-injury were the face, scalp, lips, forearm/elbow, axilla/wrist, hands/fingers, and thighs/knees. Significant differences were observed in the distribution of self-injury sites (nose, lips, genitals, and axillae/wrists) between the two genders (*p* < 0.05). The most important motivation for undertaking NSSI behaviours was to release negative emotions. The risk factors affecting NSSI behaviours were female gender (OR = 1.793), depression (OR = 1.961), anxiety (OR = 1.495), interpersonal relationship factors (OR = 1.099), academic stress factors (OR = 1.062), maternal emotional warmth (OR = 0.97), and maternal overinterference (OR = 1.036).

**Conclusions:**

The NSSI rate among junior high school students in Hainan was 28.9%, affecting girls more than boys. The form and site of self-injury between boys and girls were significantly different. The motivation for committing self-injurious behaviours was mainly to regulate bad emotions. Risk factors for NSSI behaviours included female gender, anxiety, depression, interpersonal relationship factors, academic stress factors, and maternal emotional overinterference, while maternal emotional warmth was a protective factor.

**Supplementary Information:**

The online version contains supplementary material available at 10.1186/s40359-023-01227-x.

## Background

Nonsuicidal self-injury (NSSI) refers to intentional damage to one’s own body tissues without suicidal intent, the purpose of which is not socially sanctioned [1]. NSSI can be self-perpetuating; thus, repeated NSSI behaviours deepen wounds and cause new wounds or even suicide [1–3]. According to previous epidemiologic data, NSSI is prevalent in people of all ages, among which adolescents have a very high prevalence rate [1–6]. In the US adult clinical population, the prevalence of NSSI is between 19% and 25% [7]. Of the clinical adolescent population of concern to psychologists, ≥ 40% have reported NSSI behaviours [5], while the prevalence of NSSI in the nonclinical adolescent population < 18 years of age (who do not turn to psychologists for help) is estimated to be approximately 14% [6]. In 2015, a large epidemiologic study on NSSI in the United States demonstrated that the lifetime incidence of NSSI among community adolescents was 17.6% [8]. In 2018, a global epidemiologic survey showed that the incidence of NSSI among adolescents in the past year was 19.5%, and the NSSI rate in Asian countries was significantly higher than the NSSI rate in European and American countries [9]. A meta-analysis indicated that the total incidence of NSSI per year among junior high school students from mainland China is 27.4% [10]. Another study showed that 22.1% of NSSI participants had repeated NSSI behaviour at the 1-year follow-up evaluation [11]. According to previous studies [5, 6, 8–12], NSSI is highly prevalent among adolescents at home and abroad. Therefore, NSSI is now considered a serious public health issue and has attracted considerable attention worldwide. Due to the large population base in China and the high incidence of NSSI, a large number of NSSI events are observed among Chinese adolescents. It is necessary to conduct in-depth research on NSSI among Chinese adolescents.

According to Nock’s comprehensive theoretical model, NSSI behaviour is an adaptive means to regulate aversive emotional experiences and interpersonal relationships [13]. The risk factors for NSSI in adolescents include the following: individual internal/interpersonal vulnerability (e.g., aversive emotional experience and poor emotional regulation), distal risk factors (e.g., childhood abuse, family dysfunction, and parental criticism), and proximal risk factors (e.g., negative life events) [13]. Previous literature has shown that emotional regulation is one of the most common functions of NSSI, and individuals with mood disorders are at significantly higher risk of developing NSSI [8, 14–17]. Depression and anxiety are proven risk factors for NSSI in adolescents [14–17]. The distal risk factors for NSSI are mainly related to the quality of the individual’s family and social environment. Family is the main place for individual socialization. According to attachment theory, parenting styles profoundly shape the development of adolescents’ cognitive, social, and emotional regulation functions [18, 19]. Poor parenting has been shown to be associated with a variety of psychopathological disorders, including internalized emotions (anxiety, depression, anger) and externalized behaviours (aggression, impulsive behaviour) [20, 21]. An effective parenting style can deliver more emotional warmth and support, which is conducive to the development of children’s emotional regulation ability. In contrast, a negative parenting style causes insecure parent‒child attachment, leads to emotional and social dysfunction in adolescents, and makes adolescents prone to NSSI and other negative behaviours [20, 21]. Domestic and foreign studies have shown that parental punishment, criticism and excessive control are important risk factors for the occurrence of NSSI, and positive parenting styles such as parental emotional warmth can reduce the risk of NSSI in adolescents [6, 18–24]. As a proximal risk factor for NSSI, negative life events are also closely associated with NSSI in adolescents, which causes psychological distress in individuals. According to the stress-exposure model of psychopathology, experiencing high levels of life stress is an important psychosocial factor leading to emotional problems such as depression [25, 26]. As teenagers are mentally immature and lack self-control ability, they may adopt NSSI to adjust to the negative emotions brought by pressure [27, 28]. A study on Chinese adolescents showed a significant positive correlation between stressful life events and NSSI [29].

However, there are few previous studies on the above risk factors for NSSI among Chinese adolescents. Previous domestic studies on NSSI among adolescents were mainly conducted in mainland China, while few studies have focused on NSSI behaviours among adolescents in coastal areas [10, 11, 17]. Therefore, in the present study, we analysed the current status of NSSI behaviours among junior high school students in Hainan, a coastal area in China. In addition, based on Nock’s comprehensive theoretical model, we focused on the NSSI behavioural characteristics, patterns, motivations, and risk factors in these subjects.

## Subjects and methodology

### Subjects and questionnaire survey

A questionnaire survey was administered using multistage cluster sampling in this cross-sectional study. In the first stage, three areas in Hainan Province with social, economic, and cultural differences (rural areas, towns, and cities) were selected. In the second stage, 1 ~ 2 public junior high schools were randomly selected from the three regions. In the third stage, 2–3 classes from the first-third grades of each junior high school were randomly selected to respond to questionnaires (Supplemental file 1) as part of the investigation. Finally, one junior high school was chosen from rural areas, two from towns, and two from cities. The cities had a permanent population of > 200,000 and extensive housing, transportation, health, utilities, land use, commodity production, and communication systems. Towns had a permanent population of > 2000 and < 100,000, and 50% of the population was nonagricultural. 90% of the workers lived together and mainly engaged in agricultural production in rural areas.

The survey was active for 1 month. The questionnaires were distributed to students in the first, second, and third years of junior high school on a class-by-class basis. Before the survey commenced, the guardians of the subjects signed the informed consent form; anonymity was assured. Considering the young age of the subjects, guidance on the survey was provided by two graduate students majoring in clinical psychology and one psychology professor with > 3 years of psychology work experience. Any subjects who had doubts about questions in the questionnaire could ask the graduate students and professor for help. The subjects were instructed on the method of using the scales and self-scoring. One-to-many explanations were provided on a class-by-class basis.

### Sample estimation

According to the literature, the NSSI behaviour detection rate among junior high school students in China is approximately 27.4% [10]. In the current study, the sample size was estimated according to the detection rate of 27.4%. The test level α = 0.05 was adopted, and the allowable error was 10% of the prevalence rate. The sample size of 1018 cases was calculated according to the sample size formula for simple random sampling. It was estimated that the design efficiency was 1.2, and the sample size was the sample size calculated by simple random sampling multiplied by the design efficiency (1018*1.2 = 1222). To increase the representativeness of the sample, the additional sample size was approximately 50%, so it was determined that the required sample size was approximately 1900. Sample size was calculated by the following formula:

$$N={\left[\frac{Z\alpha /2}{\delta }\right]}^{2}\rho (1-\rho),$$

where N = sample size, ρ = the expected positive rate calculated with a detection rate of 27.4%, 1 - rho = 1-0.274 = 0.726, δ = tolerance error (i.e., 10% of the expected positive rate), and Zα/2 = 1.96 when the confidence limit of the sample positive rate was 95% (α = 0.05).

### Survey tools

A self-prepared questionnaire was administered to collect the sociodemographic data of the subjects, including general information, academic performance, place of residence, and religious belief.

#### Ottawa self-injury inventory

The subjects completed a self-assessment using the Chinese version of the Ottawa Self-injury Inventory (OSI) [30]. The OSI consists of 26 items to assess the severity, behavioural characteristics, impulsivity, and source of NSSI ideas.

The 14th question on the OSI is the Ottawa Self-injury Inventory-Functions (OSI-F) item, which is used to determine the motivations for the first and present episodes of self-injury. The OSI-F evaluates various motivations for primary and sustained NSSI in subjects based on 25 items using a 5-point Likert scale (0 = never; 1 ~ 3 = sometimes; 4 = always). The 25 items that comprise the four motivation categories underlying NSSI behaviours (external emotion regulation, social influence, internal emotion regulation, and sensation-seeking functions) were examined. The internal consistency of Cronbach’s α for the four factors varied between 0.637 and 0.896. The internal consistency of the Chinese OSI-F had a Cronbach’s α = 0.952.

The 20th question of the OSI is the Ottawa Subscale of Characteristics of Self-injury Addiction item, which is used to assess NSSI addiction. The scoring criteria are consistent with the OSI-F; the internal consistency Cronbach’s α = 0.87.

The OSI scale took 15–20 min to complete.

#### Adolescent self-rating life events checklist

Another self-assessment was conducted using the Adolescent Self-rating Life Events Checklist (ASLEC) [31]. The duration examined by the evaluation depends on the purpose of the study and can be the most recent 3, 6, 9, or 12 months. This measure consists of 27 items and 6 dimensions covering interpersonal relationships, study pressure, punishment, loss, health adaptations, and others (nonspecified). For each event, the answer first determines whether the event happened within the limited time frame. If the event did not occur, a “0” is entered next to the item that did not occur. If the event did occur, a 5-point Likert scale is adopted according to the psychological feelings at the time of the event occurred: (1), mild (2), moderate (3), severe (4) or extremely severe (5). The higher the factor score is, the greater the impact of this type of life event. These six dimensions collectively explained 44% of the variability of the entire measure. The Cronbach’s alpha coefficient was 0.85. The Spearman-Brown corrected split-half reliability was 0.88. The test-retest reliability coefficient was 0.69.

The ASLEC scale took 5–10 min to complete.

#### Perceived parental rearing patterns scale

The Chinese version of the Perceived Parental Rearing Patterns Scale (Egma Minnen av Bardndosnauppforstran [EMBU]) was administered to subjects [32]. The perceived parental patterns of the subjects were assessed during the previous 12 months using the EMBU. The EMBU consists of two sections (parenting styles of the father and mother), covering 15 parental rearing patterns, as follows: abuse, parental alienation, punishment, humiliation, rejection, overprotection, overinterference, tolerance, orientation of action, attribution style, encouragement, preference for one’s siblings, preference for the subject, and nonspecific behaviours. EMBU consists of mother’s rearing patterns scale and father’s rearing patterns scale. Mother’s rearing patterns scalecan be divided into five dimensions: rejection and denial, emotional warmth, favouritism, punishment, and overprotection and interference. Father’s rearing patterns scale can be divided into six dimensions: rejection and denial, emotional warmth, favouritism, punishment, overprotection and interference. The Chinese version of the EMBU had an internal consistency reliability of 0.50–0.88, a split-half reliability of 0.50–0.82, and a test-retest reliability of 0.58–0.82. Each item was rated on a 4-point scale, depending on the frequency of events, as follows: never, occasional, often, and always. The entire EMBU scale took 15–20 min to complete.

#### Self-rating anxiety scale and self-rating depression scale

The Self-rating Anxiety Scale （SAS） and the Self-rating Depression Scale (SDS) took 5 min each to complete.

The Self-rating Anxiety Scale was administered to the subjects to evaluate their anxiety levels [33]. The scale consists of 20 statements and corresponding questions and uses a 4-point Likert scale, as follows: 1 = never or occasionally, 2 = sometimes, 3 = often, and 4 = always. Of the 20 items, 5 (5, 9, 13, 17, and 19) are scored in reverse order, and the remaining items are scored in positive order. The cumulative items are divided into the total score. The total score is multiplied by 1.25 to obtain the standard score. A score < 50 is normal, a score between 50 and 60 indicates mild anxiety, a score between 61 and 70 is moderately anxious, and a score ≥ 70 is severely anxious.

The SDS was administered to the subjects to evaluate their depression levels [34]. The scale contains 20 questions, each reflecting a depression-related symptom, but the 20 items can be divided into 4 depression-specific symptoms: (1) emotional symptoms, including depressive mood and crying; (2) physical disorders, including daytime differences in mood, sleep disorders, loss of appetite, loss of libido, weight loss, constipation, tachycardia, and fatigue; (3) psychomotor disorders, including psychomotor hysteresis and agitation (two items); and (4) psychological disorders of depression, including confusion, hopelessness, irritability, indecision, self-depreciation, emptiness, repeated thoughts of suicide, and dissatisfaction (8 items) [35].

The SDS uses a 4-point Likert scale, as follows: 1 = never or occasionally, 2 = sometimes, 3 = often, and 4 = always. Among the 20 items, 10 (2, 5, 6, 11, 12, 14, 16, 17, 18, and 20) are scored in reverse order, and the remaining 10 items are scored in positive order. The total score is the sum of the score for all items and is multiplied by 1.25 to obtain the standard score. A standard score < 50 points is normal, a score between 50 and 60 is mildly depressed, a score between 61 and 70 is moderately depressed, and a score ≥ 70 is severely depressed.

### Statistical analysis

The completed questionnaires were retrieved and reviewed, checked, and coded. A database was built using Epidate 3.1 software. Descriptive analysis was performed for the basic demographic data. A t test, one-way ANOVA, and multiple logistics regression were carried out. All of the above analyses were performed using SPSS 22.0. A p value < 0.05 was statistically significant.

## Results

### Baseline information of the samples

A total of 1900 questionnaires (380 for each school) were distributed, and 1885 completed copies were retrieved and validated for a completion rate of 99.2%. The basic demographic data of the samples are shown in Table 1. The age range of the 1885 subjects was 11–16 years, with an average age of 13.08 ± 0.911 years. There were 921 boys and 964 girls.

**Table 1:** Basic demographic data

		Frequency	Percentage	Valid percentage	Cumulative percentage
Gender	Boy	921	48.9	48.9	48.9
	Girl	964	51.1	51.1	100.0
Age	11	5	0.3	0.3	0.3
	12	533	28.3	28.3	28.5
	13	807	42.8	42.8	71.4
	14	402	21.3	21.3	92.7
	15	124	6.6	6.6	99.3
	16	14	0.7	0.7	100.0
Academic performance	Excellent	70	3.7	3.7	3.7
	Good	441	23.4	23.6	27.4
	Fair	1024	54.3	54.8	82.2
	Poor	273	14.5	14.6	96.8
	Very poor	59	3.1	3.2	100.0
Place of residence	Rural area	813	43.1	43.1	43.1
	Town	524	27.8	27.8	70.9
	City	548	29.1	29.1	100.0
Father’s education	College	397	21.1	21.5	21.5
	Technical secondary school	550	29.2	29.8	51.3
	Junior high school	751	39.8	40.7	92.0
	Primary school	148	7.9	8.0	100.0
Mother’s education	College	296	15.7	16.1	16.1
	Technical secondary school	533	28.3	29.1	45.2
	Junior high school	797	42.3	43.5	88.7
	Primary school	208	11.0	11.3	100.0

Academic performance: Taking the scores of the subjects in the final examination at the end of the term as the standard, the subjects rated themselves. The full score was 100. A score > 90 was good, 80 ~ 90 was fair, 70 ~ 80 was poor, and < 70 was very poor

### Gender, age, and geographic features of subjects committing NSSIs

The gender, age, and geographic features of subjects committing NSSIs are shown in Table 2. Five hundred forty-four subjects admitted that they had at least one NSSI episode in the past 12 months, accounting for 28.9% of all subjects surveyed. The 544 students committing NSSIs (average age, 13.08 ± 0.883 years) included 172 boys and 372 girls. Of the 544 students, 228 were from rural areas, 169 were from towns, and 147 were from cities. The NSSI rate was 18.7% among boys and 38.6% among girls in the previous 12 months. One-way ANOVA indicated that girls had significantly more NSSI behaviours than boys (*p* < 0.001). The incidence of NSSIs did not differ significantly across the age groups (*p* > 0.05) or among rural areas, towns, and cities (*p* > 0.05).

**Table 2:** Gender, age, and geographic features of subjects committing NSSIs

	NSSI Group n = 544		Self-injury ideation only Group n = 125		non-NSSI Group n = 1216		*χ* ^*2*^	*p*
	Cases (N) Percentage (%)		Cases (N) Percentage (%)		Cases (N) Percentage (%)			
**Gender**								
Boy	172	18.7	60	6.5	689	74.8	94.380	< 0.001*
Girl	372	38.6	65	6.7	527	54.7		
**Age (years)**								
11–12	150	27.9	43	8.0	345	64.1	6.720	0.348
13	236	29.2	54	6.7	517	64.1		
14	125	31.1	22	5.5	255	63.4		
15–16	33	23.9	6	4.3	99	71.7		
**Geographic region**								
Rural area	228	28.0%	50	6.2%	535	65.8%	5.049	0.282
Town	169	32.3%	35	6.7%	320	61.1%		
City	147	26.8%	40	7.3%	361	65.9%		

*P **< 0.05**

A total of 125 students had the idea of self-injury only but did not commit NSSI behaviours. The other 1216 students had neither ideas of self-injury nor NSSI behaviours.

### People informed about the NSSI episodes

According to our survey, 544 junior high school students committed 737 NSSI events in total; however, of these events, 309,309 (41.9%) were kept secret. The friends of NSSI subjects were informed about 160 events (160/737 [21.7%]) and someone else was informed about 150 events (150/737 [20.4%]). Psychologists or other types of mental health professionals were informed about 41 events (41/737 [5.6%]), family members were informed about 27 events (27/737 [3.7%]), and other individuals were informed about 50 events (50/737 [6.8%]).

### Methods and sites of self-injury

The most common method of self-injury among 544 junior high school students committing NSSIs was scratching/bruising, followed by hitting oneself, pulling out hair, biting, head banging, and cutting (Table 3). The NSSI methods of scratching/bruising, hitting oneself and cutting more commonly occurred in girls than boys (*p* < 0.05; Table 3).

**Table 3:** Gender-based differences in the self-injury method

Self-injury method	Boy		Girl		*χ* ^*2*^	*p*
	Episode	Percentage	Episode	Percentage		
	(N)	(%)	(N)	(%)		
Cutting	19	6.8	118	17.5	26.681	< 0.001*
Scratching/bruising	54	19.3	162	24.1	7.256	0.007*
Delayed wound healing	12	4.3	38	5.7	1.478	0.224
Burning	5	1.8	4	0.6	1.430	0.232
Biting	32	11.4	69	10.3	0.000	0.987
Hitting oneself	47	16.8	68	10.1	5.773	0.016*
Pulling out hair	32	11.4	55	8.2	1.277	0.258
Severe nail biting/nail injuries	14	5.0	22	3.3	0.943	0.332
Piercing the skin with sharp objects	14	5.0	34	5.1	0.146	0.702
Body puncture	0	0.0	1	0.2	Fisher	1.000
Excessive alcohol consumption	1	0.4	13	2.0	2.904	0.088
Attempting to break bones	4	1.4	4	0.6	0.553	0.457
Head banging	22	7.9	39	6.0	0.629	0.428
Taking too many medications	2	0.7	4	0.6	0.000	1.000
Not taking enough medications	0	0.0	4	0.6	0.681	0.409
Eating or drinking something that is not food	5	1.8	9	1.3	0.002	0.966
Other	17	6.1	29	4.3	0.662	0.416
Total	280	/	673	/	/	/

*P **< 0.05**

The most common sites of self-injury were the face, scalp, lips, forearm/elbow, axilla/wrist, hands/fingers, and thighs/knees (Table 4). The interaction analysis indicated significant differences in the distribution of self-injury sites (nose, lips, genitals, and axillae/wrists) between the two genders (*p* < 0.05; Table 4).

**Table 4:** Gender-based comparison of the site of self-injury

Self-injury site	Boy		Girl		*χ* ^*2*^	*p*
	Episode (N)	Percentage (%)	Episode(N)	Percentage (%)		
Scalp	21	7.3	41	6.4	0.164	0.685
Eyes	5	1.7	7	1.1	0.196	0.658
Ears	4	1.4	4	0.6	0.553	0.457
Face	30	10.4	45	7.0	2.827	0.093
Nose	8	2.8	4	0.6	5.413	0.020*
Lips	19	6.6	21	3.3	4.275	0.039*
Mouth	2	0.7	6	0.9	0.001	0.982
Neck/throat	7	2.4	20	3.1	0.426	0.513
Chest	5	1.7	3	0.5	2.279	0.131
Breasts	2	0.7	2	0.3	0.064	0.800
Back	2	0.7	6	0.9	0.001	0.982
Shoulder	7	2.4	10	1.6	0.742	0.389
Abdomen	3	1.0	7	1.1	0.000	1.000
Buttock	1	0.4	4	0.6	0.006	0.938
Genitals	6	2.1	2	0.3	5.178	0.023*
Upper arm/elbow	25	8.7	55	8.5	0.006	0.939
Lower arm/wrist	29	10.1	197	30.5	63.105	0.000*
Hands/fingers	57	19.8	112	17.3	0.505	0.477
Thighs/knees	26	9.0	41	6.4	1.826	0.177
Calves/ankles	13	4.5	32	5.0	0.169	0.681
Feet/toes	9	3.1	11	1.7	1.720	0.190
Other	7	2.4	13	2.0	0.110	0.740
Rectum	0	0.0	3	0.5	1.395	0.555
Total	288	/	646	/	/	/

*P **< 0.05**

### Motivations for self-injury

The results of the OSI were statistically analysed, and the most common reason for NSSI was to manage negative emotions (Table 5). Girls compared to boys had significantly more of the following negative emotions and related NSSI motivations: “to vent unbearable nervousness,“ “to make my parents no longer angry with me,“ “to punish myself,“ “to distract my attention from unhappy memories,“ “to release anger,“ “to relieve sadness or stop feeling down,“ “to stop thinking about killing myself,“ “to stop myself from committing suicide” “to make myself feel a sense of realness when I feel numb and unreal,“ “to release frustrated feelings,“ and “to prove how much pain I can tolerate” (all *p < 0.05*; Table 5).

**Table 5:** Motivations for self-injury

Motivations for self-injury	Boy		Girl		*χ* ^*2*^	*p*
	Episode (N)	Percentage (%)	Episode (N)	Percentage (%)		
To vent unbearable nervousness	61	35.5	118	50.5	10.765	0.001*
To seek sensation, such as taking drugs	9	5.2	31	8.3	1.660	0.198
To make my parents no longer angry with me	42	24.4	61	16.4	4.930	0.034*
To stop feeling lonely and empty	54	31.4	120	32.3	0.040	0.841
To get somebody’s attention	37	21.5	70	18.8	0.540	0.462
To punish myself	60	34.9	164	44.1	4.112	0.043*
To experience a sense of excitement	16	9.3	42	11.3	0.488	0.485
To prevent getting into trouble because of what I have done	19	11.0	36	9.7	0.243	0.622
To distract my attention from unhappy memories	60	34.9	204	54.8	18.750	0.000*
To change my body image and/or appearance	20	11.6	27	7.3	2.845	0.092
To become part of a community	20	11.6	32	8.6	1.246	0.264
To release anger	78	45.3	207	55.6	4.999	0.025*
To tell others how hurt I am	27	15.7	43	11.6	1.797	0.180
To experience the physical pain of a specific site when feeling other unbearable pain	29	16.9	73	19.6	0.589	0.443
To prevent others from having too high expectations of me	28	16.3	75	20.2	1.155	0.283
To relieve sadness or stop feeling depressed	68	39.5	250	67.2	37.080	0.000*
To stop thinking about killing myself	23	13.4	107	28.8	15.321	0.000*
To stop myself from committing suicide	19	11.0	85	22.8	10.597	0.001*
To make myself feel a sense of realness when I feel numb and unreal	19	11.0	94	25.3	14.456	0.000*
To release frustrated feelings	66	38.4	213	57.3	16.792	0.000*
To escape doing things I don’t feel like doing	36	20.9	97	26.1	1.686	0.194
To prove how much pain I can tolerate	13	7.6	53	14.2	4.937	0.026*
To experience sexual arousal	8	4.7	21	5.6	0.230	0.631
To reduce sexual arousal	11	6.4	21	5.6	0.120	0.730
Other (please specify)	5	2.9	33	8.9	6.439	0.011*
Total	828	/	2277	/	/	/

*P **< 0.05**

### Factors influencing the NSSI and non-NSSI groups

According to the rank-sum test, the scores were significantly higher in the NSSI group than in the non-NSSI group in each life event dimension: interpersonal relationships, study pressure, punishment, health adaptation and others (*p < 0.05*; Table 6), which demonstrated that negative life events greatly influenced the students.

**Table 6:** Comparison of life event dimensions between the NSSI and non-NSSI groups

Life event dimension	NSSI group	Non-NSSI group	z	P
Interpersonal relationships	6	3	17.361	< 0.001*
Study pressure	5	4	15.543	< 0.001*
Punishment	5	2	12.767	< 0.001*
Health adaptation	2	1	10.291	< 0.001*
Other	3	1	17.034	< 0.001*

*P **< 0.05**

In addition, the parental education level was significantly lower in the NSSI group than in the non-NSSI group based on the rank-sum test (*p < 0.05*; Table 7). There were also significant differences in the frequency of NSSI behaviours between subjects with varying levels of academic performance. Multiple comparisons showed a significant difference in the frequency of NSSI behaviours between subjects with good and fair academic performance. NSSI behaviours were more common in subjects with fair academic performance than in subjects with good academic performance (*p* < 0.05; Table 7).

**Table 7:** Factors influencing the NSSI and non-NSSI groups

Influencing factor	Non-NSSI group (n = 1341)		NSSI group (n = 544)		z	P
	**Cases (N) Percentage (%)**		**Cases (N) Percentage (%)**			
**Father’s education**						
College	293	21.80%	104	19.10%		
Technical secondary school	398	29.70%	152	27.90%	-2.026	0.043*
Junior high school	553	41.20%	237	43.60%		
Primary school	97	7.20%	51	9.40%		
**Mother’s education**						
College	227	16.90%	69	12.70%	-2.196	0.028*
Technical secondary school	378	28.20%	155	28.50%		
Junior high school	597	44.50%	251	46.10%		
Primary school	139	10.40%	69	12.70%		
**Academic performance**						
Excellent	49	3.70%	21	3.90%	-2.132	0.033*
Good	340	25.40%	101	18.6%		
Fair	719	53.60%	323	59.40%		
Poor	190	14.20%	83	15.30%		
Very poor	43	3.20%	16	2.90%		

*P **< 0.05**

Academic performance: Taking the scores of the subjects in the final examination at the end of the term as the standard, the subjects rated themselves. The full score was 100. A score > 90 was good, 80 ~ 90 was fair, 70 ~ 80 was poor, and < 70 was very poor

### Comparison of depression and anxiety levels between the NSSI and non-NSSI groups

The depression and anxiety levels of the NSSI and non-NSSI groups were significantly different. Depression and anxiety were more severe in the NSSI group than in the non-NSSI group (both *p* < 0.05; Table 8). The ratings of depression and anxiety were higher, and the NSSI rate was higher.

**Table 8:** Comparison of depression and anxiety levels between the NSSI and non-NSSI groups

Indicator	Non-NSSI group (n = 1341)		NSSI group (n = 544)		z	P
	Cases (N) Percentage (%)		Cases (N) Percentage (%)			
**Depression rating**					20.077	< 0.001*
Normal	972	72.5%	150	27.6%		
Mild	261	19.5%	154	28.3%		
Moderate	92	6.9%	157	28.9%		
Severe	16	1.2%	83	15.3%		
**Anxiety rating**						
Normal	1238	92.3%	323	59.40%		
Mild	80	6.00%	148	27.20%	17.328	< 0.001*
Moderate	21	1.60%	38	7.00%		
Severe	2	0.10%	35	6.40%		

*P **< 0.05**

The depression and anxiety levels also varied significantly between the genders, with greater severity in girls than boys (all *p* < 0.05; Table 9). Girls were also prone to more negative moods than boys.

**Table 9:** Comparison of depression and anxiety levels between genders

Indicator	Boy		Girl		z	P
	Cases (N) Percentage (%)		Cases (N) Percentage (%)			
**Depression rating**					10.917	< 0.001*
Normal	655	71.1%	467	48.4%		
Mild	175	19.0%	240	24.9%		
Moderate	73	7.9%	176	18.3%		
Severe	18	2.0%	81	8.4%		
**Anxiety rating**						
Normal	826	89.70%	735	76.20%		
Mild	71	7.70%	157	16.30%	7.801	< 0.001*
Moderate	17	1.80%	42	4.40%		
Severe	7	0.80%	30	3.10%		

*P **< 0.05**

### Binary logistic regression of risk factors for NSSI behaviours

Based on t tests, the scores of the NSSI group were significantly lower than those of the non-NSSI group in the dimensions of parental emotional warmth and mother’s emotional warmth (both P < 0.01, Table 10). The scores of the NSSI group were significantly higher than those of the non-NSSI group in the dimensions of father punishment, father overinterference, father rejection, father overprotection, mother overinterference, mother rejection, and mother punishment (all P < 0.01, Table 10).

**Table 10:** Comparison of parental rearing patterns between the NSSI and non-NSSI groups (x ± s)

Each dimension of the perceived parental rearing pattern scale	NSSI group	Non-NSSI group	*t*	P
Parental emotional warmth	44.6746 ± 12.30097	53.9299 ± 11.24641	15.165	< 0.001*
Father’s punishment	20.6507 ± 7.6781	16.4161 ± 5.09827	11.848	< 0.001*
Father’s overinterference	22.7794 ± 6.61706	19.2028 ± 4.85236	11.422	< 0.001*
Father’s preference for the subject	7.6581 ± 2.92333	7.9142 ± 2.93119	1.72	0.086
Father’s rejection	11.0809 ± 4.1912	8.8009 ± 2.98452	11.555	< 0.001*
Father’s overprotection	13.4522 ± 4.03182	11.7211 ± 3.55948	8.729	< 0.001*
Mother’s emotional warmth	45.4265 ± 12.41539	53.7233 ± 11.37004	13.464	< 0.001*
Mother’s overinterference	37.5956 ± 9.58754	31.9635 ± 9.58754	12.295	< 0.001*
Mother’s rejection	14.7813 ± 5.49183	11.5421 ± 5.49183	12.452	< 0.001*
Mother’s punishment	14.7702 ± 5.5726	11.7457 ± 3.79299	11.614	< 0.001*
Mother’s preference for the subject	7.7177 ± 2.90509	7.9428 ± 2.95044	1.503	0.133

*P **< 0.05**

A binary logistic regression analysis was carried out by treating gender, depression rating, anxiety level, life event dimensions, and mother’s rearing pattern. Notably, the father’s rearing pattern was strongly correlated with the mother’s rearing pattern, and the former dramatically interfered with the statistical results. Therefore, only the mother’s rearing pattern was included in the regression analysis, with academic performance as an independent variable and NSSI behaviour as the dependent variable. Female gender, anxiety level, depression level, interpersonal relationships, study pressure, mother’s emotional warmth, and mother’s overinterference predicted NSSI episodes longitudinally (Table 11).

**Table 11:** Binary logistic regression of risk factors for NSSI behaviours

	B	Standard deviation	Wald	Degree of freedom	Significance level	OR	95% confidence interval of OR	
							Lower bound	Upper bound
Depression rating	0.673	0.085	63.223	1	0	1.961	1.661	2.315
Anxiety level	0.402	0.133	9.129	1	0.003	1.495	1.152	1.94
Interpersonal relationship	0.094	0.024	15.218	1	0	1.099	1.048	1.152
Study pressure	0.06	0.024	5.983	1	0.014	1.062	1.012	1.114
Female	0.584	0.129	20.567	1	0	1.793	1.393	2.308
Mother’s emotional warmth	-0.03	0.005	31.244	1	0	0.97	0.96	0.981
Mother’s overinterference	0.036	0.008	20.66	1	0	1.036	1.021	1.053
Constant	-3.487	0.49	50.727	1	0	0.031		

## Discussion

### NSSI rate and characteristics of NSSI behaviours

Five hundred forty-four subjects admitted that they had at least one NSSI episode in the previous 12 months, accounting for 28.9% of all subjects surveyed. This incidence agreed with the overall NSSI rate (27.4%) reported from an epidemiologic survey among high school students in mainland China in 2021 [10]. In addition, 125 subjects had self-injury ideations, constituting another population at higher risk for NSSIs. These subjects may continue to commit NSSIs without timely psychological intervention and help [36].

We reported a higher frequency of NSSI behaviours in girls than boys, which agreed with the findings from other studies [11, 39, 40]. Several previous studies have reported gender-related differences in NSSI surveys using different samples and found that the probability of females committing NSSIs is higher than that of males [2, 8, 11, 22, 37, 39]. Such a prevalent gender-related difference can be attributed to the fact that NSSI is a way to eliminate negative emotions [37]. Emotional disorders, such as anxiety and depression, are also factors that influence NSSI behaviours [41, 42]. According to studies at home and abroad, mood disorders, such as anxiety and depression, are more prevalent in females than males [43]. Therefore, girls commit NSSI more commonly than boys.

Our study found that junior high school students committing NSSIs were 11–16 years of age, with an average age of 13.08 ± 0.911 years, which is consistent with a previous report that the average age of the first NSSI was 11–15 years [44].

### Methods and sites of NSSIs

The most common method of NSSI was scratching/bruising, followed by hitting oneself, pulling out hair, biting, head banging, and cutting in the sample population. The NSSI methods of scratching/bruising, hitting oneself and cutting more commonly occurred in girls than boys. Some researchers have suggested that cutting is the most common method of self-injury, but biting and hitting oneself are also common [14, 17, 40]. Most studies involving NSSIs agree that cutting is the most common method of self-injury among females. Males, in contrast, prefer more violent ways to harm themselves, such as hitting, burning, or banging one part of the body [6, 14, 40]. The above disagreement in study findings might be explained by the difference in the proportion of males and females committing self-injury. In the current study, girls committing NSSIs outnumbered their male counterparts, which is similar to a previous report [38]. Some females may harm themselves in a conspicuous way to attract attention from more people and hence to obtain emotional support [5, 6, 8, 39, 45].

Unlike studies on self-injury methods, few studies have included self-injury sites. In this study, the most common sites of self-injury were the face, scalp, lips, forearm/elbow, axilla/wrist, hands/fingers, and thighs/knees. Significant differences in the distribution of self-injury sites (nose, lips, genitals, and axillae/wrists) were found between the two genders. Boys were more likely to injure their genitals than girls, which agrees with the findings from a previous study [46].

### Motivations and risk factors for NSSI behaviours

Our survey proved that releasing negative emotions is the most common motivation for NSSIs, which agreed with previous studies involving NSSIs at home and abroad [14, 16, 17]. Considering and using NSSIs as a strategy to regulate emotions is highly correlated with emotional instability and dysregulation. For example, Ross et al. [47] administered a survey to 400 adolescents and showed that those committing NSSIs were more likely to have defects in emotion recognition and integration. Biologically, some parts of the brain change due to rapid physical development during adolescence. Such changes are usually related to cognitive functions, especially emotional regulation [48]; however, the ability to control behaviours and regulate emotions (i.e., executive function) usually matures at varying rates on the physiologic scale. Neurologic development related to behavioural control takes place at a much lower rate. The above two facts jointly explain the weaker ability to control impulsive behaviours and emotions among adolescents [9, 47, 48].

The percentage of girls committing NSSIs was significantly higher than that of boys, and gender was a risk factor for NSSI behaviours in adolescents. The above finding agreed with other studies at home and abroad [2, 8, 14, 39, 49]. It has been established that the oestrogen level increases significantly in adolescent girls, resulting in higher emotional reactivity. Therefore, adolescent girls are more likely to have violent mood swings than their male counterparts [8, 27, 43, 52].

The mainstream theories relating to NSSIs generally hold that negative emotions are among the most important factors inducing NSSIs. Multivariate logistic regression showed that depression and anxiety symptom scores were positively correlated with NSSI behaviours. Depression and anxiety are proven risk factors for NSSIs in adolescents. The above results agreed with other studies at home and abroad [14–17, 49, 50].

The correlations between anxiety and depression and NSSI behaviours in adolescents can be explained by the mainstream theories related to NSSIs. Bentley and Nock [37] proposed a four-function model of NSSIs that suggests that the most important intrinsic motivation to trigger NSSI behaviours is to alleviate the intense negative emotions deep inside. It has been emphasized by other studies that according to the experiential avoidance model, NSSI is an avoidance behaviour that enables individuals to avoid unwanted emotions, thoughts, memories, and/or somatic sensations and to narrow attention to immediate sensations [51].

Notably, our gender-based analysis of negative emotions showed that girls had higher anxiety and depression levels than boys. Our results agreed with those on the gender-based differences in emotional functions at home and abroad. Compared with males, females are more likely to internalize external pressures into their own emotional disturbance, resulting in negative emotions, such as anxiety and depression [25, 27, 53, 45]. Higher levels of pressure from interpersonal relationships or other life events may be closely related to an increase in the frequency of NSSI episodes and expectations about depression symptoms among females [25].

Life events refer to social experiences or changes with a specific onset and course that have a psychological impact on the individual. Previous studies have demonstrated that negative life events may serve as proximal risk factors for NSSIs and can be used to predict NSSI episodes [26–28, 54]. The NSSI group scored significantly higher in all life event dimensions than the non-NSSI group. Further analysis showed that for the NSSI group, the life event dimensions of interpersonal relationships, study pressure, and something else were positively correlated with NSSI behaviours. These results agreed with other studies at home and abroad [26–29, 54]. According to the Bentley and Nock four-function model, NSSI behaviours are a maladaptive coping strategy for individuals to adjust interpersonal relationships or avoid interpersonal pressure [38]. Given the psychological immaturity of adolescents, they may resort to NSSI to cope with conflicts with parents or peers when they feel interpersonal pressure [23, 38].

The parental rearing pattern refers to a fixed mode of action that can be perceived in parents rearing their children. The results of this survey showed that mothers’ emotional warmth was a protective factor for NSSIs, while mothers’ overinterference was a risk factor for NSSIs. Negative parental rearing patterns are considered important risk factors for NSSIs, while positive parental rearing patterns reduce the incidence of NSSIs among adolescents [4, 5, 22, 24, 55, 56]. It has been shown that parents play a crucial role in guiding children’s emotional socialization. According to family invalidation theory, parental rearing patterns lie at the core of family invalidation. Negative parental rearing patterns, such as punishment, neglect, criticism, and denial, may result in failure to respond to children’s basic emotional needs. Children brought up in such a family environment may be unable to express their emotions via healthy pathways. Rather, children’s ability to regulate and express their emotions will be repressed, and their emotional regulation function tends to become pathologic [57]. Adolescents growing up in an invalidating family are vulnerable to emotional and cognitive defects and usually fail to adopt adaptive coping strategies in the face of overwhelmingly negative emotions but resort to NSSI [22, 56]. As analysed above, NSSI behaviours may be the result of specific parental rearing patterns.

This study has provided new information on the risk factors associated with NSSIs by investigating the influence of internal and external factors so that parents, teachers, and psychological professionals can better understand the epidemiologic characteristics of adolescent nonsuicidal NSSIs and the possible causes of NSSIs. In addition, our study has provided a reference for early prevention and intervention measures for nonsuicidal NSSIs. Additionally, to be clear, this was not a clinical study because the subjects were adolescents in a school setting, not patients in a health care setting.

### Limitations

NSSI behaviours are usually concealed and associated with a sense of shame. These reasons might have resulted in a low exposure rate of NSSI behaviours. The prevalence of NSSIs might have been underestimated in the present study. In addition, all of the scales used were self-rating scales, and the subjects were asked to recall whether they had committed NSSIs in the past 12 months. In this case, report and recall biases were inevitable. Finally, all samples were collected from multiple centres located in the same geographic region, and the findings may not apply to other geographic regions.

## Conclusion

The NSSI rate among junior high school students in Hainan was 28.9%, affecting girls more than boys. The form and site of self-injury between boys and girls were significantly different. The motivation for committing self-injurious behaviours was mainly to regulate bad emotions. Risk factors for NSSI behaviours included female gender, anxiety, depression, interpersonal relationship factors, academic stress factors, and maternal emotional overinterference, while maternal emotional warmth was a protective factor.

## Electronic supplementary material

Below is the link to the electronic supplementary material.

Supplementary Material 1

## Data Availability

All data analysed during this study are included in this published article.

## List of abbreviations

ASLEC: Adolescent Self-rating Life Events Checklist
NSSI: Nonsuicidal self-injury
OSI-F: Ottawa Self-injury Inventory-Functions
OSI: Ottawa Self-injury Inventory
