# Supplementary material for: Current status of nonsuicidal injuries and associated factors among junior high school students in Hainan Province, China: a cross-sectional study
Source: BMC Psychol. 2023 Jul 5;11:199. doi: 10.1186/s40359-023-01227-x (PMC10324120; doi:10.1186/s40359-023-01227-x)
Supplement: Supplementary file 1 — Supplementary Material 1 [file 40359_2023_1227_MOESM1_ESM.docx]

**Dear students: Hello! Welcome to participate in the mental health survey of middle school students. The survey results are only regarded as academic research Use, will not cause any impact on you, all your choices are not right or wrong, please fill in according to their own real situation answer. Thank you for your support and cooperation!**

**First, please fill in the following personal information carefully.**

1.Age: ____ 2. Sex: ____ 3. Ethnicity: ____ 4. Grade: ______ 5. School: ___________

6. Your academic performance: very good □ good □ generally □ poor □ very poor □ (make a choice in □)

7. You are from: rural □ township □ city □ 8. Are you the only child: Yes □ No □

9. Are you a student leader: Yes□ No □ 10. If there is religious belief: Religious sect______ Religious time: _____ years

Questionnaire beginning: To ensure the validity of the questionnaire, please read the instructions of the questionnaire carefully and complete all the questions (5 questionnaires).

**Questionnaire 1:**

| **How often in** **the** **past** **month** **have** **you:**  *circle* *“0”* *if* *not* *at* *all* *and* *circle* *“3”* *if* *daily* | Not at all | At least once | weekly | daily |
| --- | --- | --- | --- | --- |
| **Thought about injuring yourself without the intention to kill yourself?** | 0 | 1 | 2 | 3 |
| **Actually injured yourself, without the intention to kill yourself?** | 0 | 1 | 2 | 3 |

| **How** **often** **in** **the** **past 6 months have** **you:**  *circle* *“0”* *if not* *at* *all* *and* *circle* *“4”* *if* *daily* | Not at all | 1 to 5 times | monthly | weekly | daily |
| --- | --- | --- | --- | --- | --- |
| **Thought about injuring yourself without the intention to kill yourself?** | 0 | 1 | 2 | 3 | 4 |
| **Actually injured yourself, without the intention to kill yourself?** | 0 | 1 | 2 | 3 | 4 |


| **How** **often** **in** **the** **past** **year** **have** **you:**  *circle* *“0”* *if* *not* *at* *all* *and* *circle* *“4”* *if* *daily* | Not at all | 1 to 5 times | monthly | weekly | daily |
| --- | --- | --- | --- | --- | --- |
| **Thought about taking your life (killing yourself)?** | 0 | 1 | 2 | 3 | 4 |

**4. Have you ever made an actual attempt to take your life?** no□ yes□

If yes, then please indicate the number of times:

in the past month: ___ in the past 6 months: ___ in the past year: ___ prior to one year ago: ___

1. **Have you ever been treated by a doctor after injuring yourself on purpose? (e.g., stitches, wound dressings, etc.)** no□ yes□

If yes, how often did a doctor treat you in the past year for hurting yourself on purpose?_______ time(s)

1. **Have you been kept in hospital because of hurting yourself on purpose?** no□ yes□

If yes, how many times in the *past year* did you stay overnight in emergency? ______________

If yes, how many times in the *past year* did you get admitted to a hospital unit?______________

1. **If you indicated that you had thought about or actually injured yourself in questions 1-4:**

How old were you when you started to self-injure? ___________________ (years old)

1. **The first time you hurt yourself, where did you get the idea?** (please √ only one)

□I read about it on an internet website

□I read about it on a Web Blog

□I read about it in a book or magazine

□I saw it happen in a movie or on television

□I saw other people do it in a non-hospital setting

□I heard about it from other people in a non-hospital setting

□I heard about it from other people in a hospital setting

□I saw other people do it in a hospital setting

□It was my own idea

□Other (please list)_________________________

| **When** **you** **get** **the** **urge** **to** **hurt yourself:**  *circle* *“0”* *if* *not* *at* *all* *and* *circle* *“4”* *if* *extremely* | **Not at all** |  | **somewhat** |  | **extremely** |
| --- | --- | --- | --- | --- | --- |
| The urge is distressing / upsetting | 0 | 1 | 2 | 3 | 4 |
| The urge is comforting | 0 | 1 | 2 | 3 | 4 |
| The urge is intrusive / invasive | 0 | 1 | 2 | 3 | 4 |

1. **Do you only harm yourself after taking drugs or alcohol?** no□ yes□
2. **Do you let other people know that you harm yourself?**

□no one

who do you tell?

□some people

□most people

□friend(s)

□psychologist/psychiatrist

□other Mental Health Professional

□telephone helpline

□family member(s)

□family doctor

□school counsellor

□other (specify)

1. **a) What areas of your body did/do you injure?**

Please **(√)** all that apply

|  | WHEN YOU  FIRST  STARTED | CURRENTLY  (past month  if still self-  injuring) |
| --- | --- | --- |
|  |  |  |
|  |  |  |
|  |  |  |
| Scalp |  |  |
| Eye(s) |  |  |
| Ear(s) |  |  |
| Face |  |  |
| Nose |  |  |
| Lips |  |  |
| Inside of mouth |  |  |
| Neck/throat |  |  |
| Chest |  |  |
| Breast(s) |  |  |
| Back |  |  |
| Shoulder(s) |  |  |
| Abdomen |  |  |
| Hips/buttock(s) |  |  |
| Genitals |  |  |
| Rectum |  |  |
| Upper arm/elbow |  |  |
| Lower arm/wrist |  |  |
| Hand/fingers |  |  |
| Thigh/knee |  |  |
| Lower leg/ankle |  |  |
| Foot/toes |  |  |
| Other (specify): |  |  |

b) Above, please circle the part that you currently injure the most.

1. **a) How did/do you injure yourself (without meaning to kill yourself)?**

Please **(√)** all that apply

|  | WHEN  YOU  FIRST  STARTED | CURRENTLY  (past month  if still self-  injuring) |
| --- | --- | --- |
|  |  |  |
|  |  |  |
|  |  |  |
| Cutting |  |  |
| Scratching |  |  |
| Interfering with wound healing |  |  |
| Burning |  |  |
| Biting |  |  |
| Hitting |  |  |
| Hair pulling |  |  |
| Severe nail biting and/or nail injuries |  |  |
| Piercing skin with sharp pointy objects |  |  |
| Piercing of body parts |  |  |
| Excessive use of street drugs |  |  |
| Excessive use of alcohol |  |  |
| Trying to break bones |  |  |
| Headbanging |  |  |
| Taking too much medication |  |  |
| Taking too little medication |  |  |
| Eating or drinking things that are not food |  |  |
| Other (specify): |  |  |

b) Above, please circle the method that you currently use the most

| **Why do you think you started and if you continue, why do you still self-injure (without meaning to kill yourself)?**  *Please circle the number that best represents how much your self-injury is due to that reason. Circle “0” if it has never been a reason that you self-injure and “4” if it has always been a reason that you self-injure.* | **Why did you start?** | | | **If you continue, why do you continue?** | | |
| --- | --- | --- | --- | --- | --- | --- |
|  | **Never a reason** | **Sometimes a reason** | **Always a reason** | **Never a reason** | **Sometimes a reason** | **Always a reason** |
| 1. to release unbearable tension | 0 1 2 3 4 | | | 0 1 2 3 4 | | |
| 2. to experience a “high” that feels like a drug high | 0 1 2 3 4 | | | 0 1 2 3 4 | | |
| 3. to stop my parents from being angry with me | 0 1 2 3 4 | | | 0 1 2 3 4 | | |
| 4. to stop feeling alone and empty | 0 1 2 3 4 | | | 0 1 2 3 4 | | |
| 5. to get care or attention from other people | 0 1 2 3 4 | | | 0 1 2 3 4 | | |
| 6. to punish myself | 0 1 2 3 4 | | | 0 1 2 3 4 | | |
| 7. to provide a sense of excitement that feels exhilarating | 0 1 2 3 4 | | | 0 1 2 3 4 | | |
| 8. to avoid getting into trouble for something I did | 0 1 2 3 4 | | | 0 1 2 3 4 | | |
| 9. to distract me from unpleasant memories | 0 1 2 3 4 | | | 0 1 2 3 4 | | |
| 10. to change my body image and/or appearance | 0 1 2 3 4 | | | 0 1 2 3 4 | | |
| 11. to belong to a group | 0 1 2 3 4 | | | 0 1 2 3 4 | | |
| 12. to release anger | 0 1 2 3 4 | | | 0 1 2 3 4 | | |
| 13. to show others how hurt or damaged I am | 0 1 2 3 4 | | | 0 1 2 3 4 | | |
| 14. to experience physical pain in one area, when the other pain I feel is unbearable | 0 1 2 3 4 | | | 0 1 2 3 4 | | |
| 15. to stop people from expecting so much from me | 0 1 2 3 4 | | | 0 1 2 3 4 | | |
| 16. to relieve feelings of sadness or feeling “down” | 0 1 2 3 4 | | | 0 1 2 3 4 | | |
| 17. to stop me from thinking about ideas of killing myself | 0 1 2 3 4 | | | 0 1 2 3 4 | | |
| 18. to stop me from acting out ideas of killing myself | 0 1 2 3 4 | | | 0 1 2 3 4 | | |
| 19. to produce a sense of being real when I feel numb and “unreal” | 0 1 2 3 4 | | | 0 1 2 3 4 | | |
| 20. to release frustration | 0 1 2 3 4 | | | 0 1 2 3 4 | | |
| 21. to get out of doing something that I don’t want to do | 0 1 2 3 4 | | | 0 1 2 3 4 | | |
| 22. to prove to myself how much I can take | 0 1 2 3 4 | | | 0 1 2 3 4 | | |
| 23. for sexual excitement | 0 1 2 3 4 | | | 0 1 2 3 4 | | |
| 24. to diminish feeling of sexual arousal | 0 1 2 3 4 | | | 0 1 2 3 4 | | |
| 25. other (please specify): | 0 1 2 3 4 | | | 0 1 2 3 4 | | |

| **If you indicated that you had thought about or actually injured yourself in questions 1-4, do you feel relief (better)**  **after harming yourself?**  *circle “0” if never and circle “4” if always* | **Never Sometimes Always** |
| --- | --- |
|  | 01234 |

If you feel relief, how long does the relief last? (please **(√)** only one)

□less than 1 minute

□1 to 5 minutes

□6 to 30 minutes

□hours

□31 to 60 minutes

□days

1. **Once you think about harming yourself, do you always do it?**  □yes □no
2. **When you hurt yourself on purpose, on average, how much time goes by between thinking about it and doing it?** (Please **(√)** 1 item only)

□less than 1 minute

□1 minute to 5 minutes

□6 minutes to 30 minutes

□over 30 minutes but less than 1 hour

□hours

□days

| **Do** **you** **feel** **physical** **pain** **when** **you** **harm** **yourself?**  *circle “0” if never and circle “4” if always* | **Never Sometimes Always** |
| --- | --- |
|  | 01234 |

| **Do you hurt or think about hurting yourself after stressful things happen?**  *circle “0” if never and circle “4” if always* | **Never Sometimes Always** |
| --- | --- |
|  | 01234 |

**If you indicated that you thought about or actually injured yourself in questions 1-4, what kinds of stressful situation(s) typically led to self-injury?**

□abandonment (please specify)_________________________________________________________

□failure (please specify)______________________________________________________________

□loss (please specify)_________________________________________________________________

□rejection (please specify)_____________________________________________________________

□other(please specify)________________________________________________________________

| **Since you started to self-injure, have you found that:**  *circle “0” if never and circle “4” if always* | **Never Sometimes Always** |
| --- | --- |
| 1.The self-injurious behaviour occurs more often than intended? | 01234 |
| 2. The severity in which the self-injurious behaviour occurs has increased (e.g., deeper cuts, more extensive parts of your body)? | 01234 |
| 3. If the self-injurious behaviour produced an effect when started, you now need to self-injure more frequently or with greater intensity to produce the same effect? | 01234 |
| 4. This behaviour or thinking about it consumes a significant amount of your time (e.g., planning and thinking about it, collecting and hiding | 01234 |
| 5. Despite a desire to cut down or control this behaviour, you are unable to do so? | 01234 |
| 6. You continue this behaviour despite recognizing that it is harmful to you physically and/or emotionally? | 01234 |
| 7. Important social, family, academic or recreational activities are given up or reduced because of this behaviour? | 01234 |

| **If** **you** **are** **trying** **to** **resist** **hurting** **yourself,** **what** **do** **you** **do** **instead?** *Please* ***(√)*** *all* *that* *apply* | |
| --- | --- |
| Never try to resist |  |
| Talk with someone |  |
| Exercise / sports |  |
| Reading writing, music, dance |  |
| Watch television, play video or computer games |  |
| Do things to relax (e.g., hot bath, yoga, deep breathing) |  |
| Use alcohol and or street drugs |  |
| Do anything to keep hands busy |  |
| Other (specify): |  |

b) For question 21, please circle the most helpful thing you do to resist hurting yourself.

| **Do you feel physical pain when you harm yourself?**  *circle “0” if never and circle “4” if always* | **Never Sometimes Always** |
| --- | --- |
|  | 01234 |

1. **What treatment(s) if any, have you received with the goal of reducing and/or eliminating your self- harm?***(Please* ***(√)*** *all items that apply)*

□I have not had treatment

□individual therapy

□family therapy

□I declined treatment

□school counselling

□medication (please specify)

□Self help (e.g., self-help books, internet)

□group therapy

other(please specify)**___________________________________________________________________**

1. **What** **treatment(s)** **if** **any,** **have** **you** **found** **the** **most** **helpful** **in** **reducing** **and/or eliminating** **your** **self-** **harm?***(Please* ***(√)*** *all* *items* *that* *apply)*

□I have not had treatment

□individual therapy

□family therapy

□I declined treatment

□school counselling

□medication (please specify)

□Self help (e.g., self-help books, internet)

□group therapy

other(please specify)___________________________________________________________________

| **I feel that this questionnaire has fully described my**  **experience of Self-Injury** | Strongly Disagree | Somewhat Agree | Strongly Agree |
| --- | --- | --- | --- |
|  | 01234 | | |

1. **Is there anything else you would like to share with us regarding your self-injury behaviour?**

**__________________________________________________________________________________________________________________________________________________________________________________________________________________________________________________________________________________________________________________________________________________________________________________________________________________________________________________________________________________________________________________**

**Questionnaire 2 instruction:** There are 20 words, each word after four squares, please read each carefully, and then according to your actual feeling in the last week, （√）in the appropriate box. At present, please choose the main emotional and somatic symptoms according to the degree of conscious symptoms.

**Answer: A no or little time; B small time; C considerable time; D most or all of the time.**

| **Items** | **A** | **B** | **C** | **D** |
| --- | --- | --- | --- | --- |
| 1. I feel down-hearted and blue. |  |  |  |  |
| 2. Morning is when I feel the best. |  |  |  |  |
| 3. I have crying spells or feel like it. |  |  |  |  |
| 4. I have trouble sleeping at night. |  |  |  |  |
| 5. I eat as much as I used to. |  |  |  |  |
| 6. I stlll enjoy sex. |  |  |  |  |
| 7. I notice that I am losing weight. |  |  |  |  |
| 8. I have trouble with constipation. |  |  |  |  |
| 9. My heart beats faster than usual. |  |  |  |  |
| 10. I get tired for no reason. |  |  |  |  |
| 11. My mind is as clear as it used to be. |  |  |  |  |
| 12. I find it easy to do the things I used to. |  |  |  |  |
| 13. I am restless and can't keep still. |  |  |  |  |
| 14. I feel hopeful about the future. |  |  |  |  |
| 15. I am more irritable than usual. |  |  |  |  |
| 16. I find it easy to make decisions. |  |  |  |  |
| 17. I feel that I am useful and needed. |  |  |  |  |
| 18. My life is pretty full. |  |  |  |  |
| 19. I feel that others would be better off if I were dead. |  |  |  |  |
| 20. I still enjoy the things I used to do. |  |  |  |  |

**Questionnaire 3 instruction:** There are 20 words, each word after four squares, please read each carefully, and then according to your actual feeling in the last week, （√）in the appropriate box. At present, please choose the main emotional and somatic symptoms according to the degree of conscious symptoms.

**Answer: A:no or little time; B:small time; C:considerable time; D:most or all of the time.**

| **Items** | **A** | **B** | **C** | **D** |
| --- | --- | --- | --- | --- |
| 1. I feel more nervous and anxious than usual. |  |  |  |  |
| 2. I feel afraid for no reason at all. |  |  |  |  |
| 3. I get upset easily or feel panicky. |  |  |  |  |
| 4. I feel like I'm falling apart and going to pieces. |  |  |  |  |
| 5. I feel that everything is all right and nothing bad will happen. |  |  |  |  |
| 6. My arms and legs shake and tremble. |  |  |  |  |
| 7. I am bothered by headaches,neck and back pains. |  |  |  |  |
| 8. I feel weak and get tired easily. |  |  |  |  |
| 9. I feel calm and can sit still easily. |  |  |  |  |
| 10. I can feel my heart beating fast. |  |  |  |  |
| 11. I am bothered by dizzy spells. |  |  |  |  |
| 12. I have fainting spells or feel like it. |  |  |  |  |
| 13. I can breathe in and out easily . |  |  |  |  |
| 14. I get feelings of numbness and tingling in my fingers,toes. |  |  |  |  |
| 15. I am bothered by stomachaches or indigestion. |  |  |  |  |
| 16. I have to empty my bladder often. |  |  |  |  |
| 17. My hands are usually dry and warm. |  |  |  |  |
| 18. My face gets hot and blushes. |  |  |  |  |
| 19. I fall asleep easily and get a good night's rest. |  |  |  |  |
| 20. I have nightmares. |  |  |  |  |

**Questionnaire 4 instruction:**Have you and your family experienced the following events in the past 12 months? Please read each of the following items carefully, if an event occurred, and check the B-E box according on the degree of distress the event causes to you. If an event does not occur, play in the A square.

**Names of Life event：A: No; B: mild； C: moderate； D:severe； E: extremely heavy**

| **Names of life events** | **A** | **B** | **C** | **D** | **E** |
| --- | --- | --- | --- | --- | --- |
| 1.Misunderstood or blamed |  |  |  |  |  |
| 2.Discrimination or cold reception |  |  |  |  |  |
| 3.Exam failure or poor grades |  |  |  |  |  |
| 4.Disputes with classmates or friends |  |  |  |  |  |
| 5.Significant changes in lifestyle |  |  |  |  |  |
| 6.I don't like to go to school |  |  |  |  |  |
| 7.Unsuccessful love or broken love |  |  |  |  |  |
| 8.A Long-term away from family members cannot be reunited |  |  |  |  |  |
| 9.Heavy learning burden |  |  |  |  |  |
| 10.Tension with a teacher |  |  |  |  |  |
| 11.I am seriously ill |  |  |  |  |  |
| 12.Relatives and/or friends are seriously ill |  |  |  |  |  |
| 13.Death of relatives and/or friends |  |  |  |  |  |
| 14.Stolen or lost things |  |  |  |  |  |
| 15.Lose face in public |  |  |  |  |  |
| 16.Family financial difficulties |  |  |  |  |  |
| 17.There are conflicts within the family |  |  |  |  |  |
| 18.Expected selections (such as various evaluations) failed |  |  |  |  |  |
| 19.Criticized or punished |  |  |  |  |  |
| 20.Transfer or suspension |  |  |  |  |  |
| 21.fined |  |  |  |  |  |
| 22.Academic pressure |  |  |  |  |  |
| 23.Fight with people |  |  |  |  |  |
| 24.Beaten and scolded by parents |  |  |  |  |  |
| 25.Family puts pressure on you to study |  |  |  |  |  |
| 26.Unexpected fright or accident |  |  |  |  |  |
| 27.Other setbacks |  |  |  |  |  |

**Questionnaire 5 instruction:**Parental parenting style is crucial to the development and growth of their children. It's hard to remind you of every detail your parents told you as a child, but each of us was impressed by the way our parents treated us as we grew up. To answer this questionnaire is to try to recall the impressions you made as a child. The questionnaire has many groups of questions, each with 1 (never), 2 (occasionally), 3 (often), and 4 (always) in four levels of answers. Please choose the most suitable level for your father and your mother, and only one answer for each question. Your father and mother may raise you the same or differently. Please answer separately, truthfully and realistically. If your parents are not complete when you are young, you can only answer the father or mother column. If it is an only child and no brothers or sisters, the relevant questions can not be answered. Please answer truthfully. Here are some examples of how to answer each question.

1. Do your parents often call you up? Never Occasionally Often Always

Father ① 2 3 4

Mother 1 2 ③ 4

1. Are your parents very affectionate to you? Never Occasionally Often Always

Father 1 ② 3 4

Mother 1 ② 3 4

Please answer：

Gender： □male □ female

Age：

You live with your parents until you are years old.

Whether the father is alive? □yes □no

(Or died when you were years old)

Whether the mother is alive? □yes □no

(Or died when you were years old)

Whether the parents are divorced? □yes □no

Divorce at your years old.

Father's education：□University (above university, junior college)□Technical secondary school (including high school）□Junior middle school □Primary school

Father's career： □Worker □Farmer □Intellectual □Cadre

Maternal education：□University (above university, junior college)□Technical secondary school (including high school）□Junior middle school □Primary school

Mother's career： □Worker □Farmer □Intellectual □Cadre

| **Items** |  | Never | Occasionally | Often | Always |
| --- | --- | --- | --- | --- | --- |
| 1. I feel my parents interfere in everything I do. | Father | 1 | 2 | 3 | 4 |
|  | Mother |  |  |  |  |
| 2. I can feel that my parents like me very much through their words and expressions. | Father | 1 | 2 | 3 | 4 |
|  | Mother |  |  |  |  |
| 3. My parents dote on me more than my brothers and sisters. | Father | 1 | 2 | 3 | 4 |
|  | Mother |  |  |  |  |
| 4. I can feel my parents love me. | Father | 1 | 2 | 3 | 4 |
|  | Mother |  |  |  |  |
| 5. My parents punished me even for small mistakes. | Father | 1 | 2 | 3 | 4 |
|  | Mother |  |  |  |  |
| 6. My parents always try to influence me imperceptibly to make me an outstanding person. | Father | 1 | 2 | 3 | 4 |
|  | Mother |  |  |  |  |
| 7. I think my parents allow me to be unique in some ways. | Father | 1 | 2 | 3 | 4 |
|  | Mother |  |  |  |  |
| 8. My parents can give me things my other brothers and sisters can't. | Father | 1 | 2 | 3 | 4 |
|  | Mother |  |  |  |  |
| 9. My parents punished me fairly and appropriately. | Father | 1 | 2 | 3 | 4 |
|  | Mother |  |  |  |  |
| 10. I think my parents are very strict with me. | Father | 1 | 2 | 3 | 4 |
|  | Mother |  |  |  |  |
| 11. My parents always dictate what I should wear or how I should dress. | Father | 1 | 2 | 3 | 4 |
|  | Mother |  |  |  |  |
| 12. My parents didn't allow me to do things that other kids could do because they were afraid something would happen to me. | Father | 1 | 2 | 3 | 4 |
|  | Mother |  |  |  |  |
| 13. When I was young, my parents used to beat me or scold me in front of others. | Father | 1 | 2 | 3 | 4 |
|  | Mother |  |  |  |  |
| 14. My parents always pay close attention to what I do at night. | Father | 1 | 2 | 3 | 4 |
|  | Mother |  |  |  |  |
| 1. when I encounter something unsatisfactory, I can feel that my parents are trying to encourage me, so that I get some comfort. | Father | 1 | 2 | 3 | 4 |
|  | Mother |  |  |  |  |
| 16. My parents always worry too much about my health. | Father | 1 | 2 | 3 | 4 |
|  | Mother |  |  |  |  |
| 17. My parents often punish me more than I deserve. | Father | 1 | 2 | 3 | 4 |
|  | Mother |  |  |  |  |
| 18. My parents get angry if I don't do what I'm told at home. | Father | 1 | 2 | 3 | 4 |
|  | Mother |  |  |  |  |
| 19. If I do something wrong, my parents always look sad, so that I have a sense of guilt, or guilt. | Father | 1 | 2 | 3 | 4 |
|  | Mother |  |  |  |  |
| 20. I find my parents difficult to approach. | Father | 1 | 2 | 3 | 4 |
|  | Mother |  |  |  |  |
| 1. My parents used to nag me about something I said or did in front of others, which made me feel very embarrassed. | Father | 1 | 2 | 3 | 4 |
|  | Mother |  |  |  |  |
| 22. I think my parents like me more than my brothers and sisters. | Father | 1 | 2 | 3 | 4 |
|  | Mother |  |  |  |  |
| 23. In meeting my needs, my parents are very stingy. | Father | 1 | 2 | 3 | 4 |
|  | Mother |  |  |  |  |
| 24. My parents often care about my score. | Father | 1 | 2 | 3 | 4 |
|  | Mother |  |  |  |  |
| 25. If faced with a difficult task, I can feel the support from my parents. | Father | 1 | 2 | 3 | 4 |
|  | Mother |  |  |  |  |
| 26. I am often used as a "scapegoat" or "black sheep" at home. | Father | 1 | 2 | 3 | 4 |
|  | Mother |  |  |  |  |
| 27. Parents are always picky about the friends I like. | Father | 1 | 2 | 3 | 4 |
|  | Mother |  |  |  |  |
| 28. arents always think that their unhappiness is caused by me. | Father | 1 | 2 | 3 | 4 |
|  | Mother |  |  |  |  |
| 29. My parents always try to encourage me, so that I become the best. | Father | 1 | 2 | 3 | 4 |
|  | Mother |  |  |  |  |
| 30. My parents always show me that they love me. | Father | 1 | 2 | 3 | 4 |
|  | Mother |  |  |  |  |
| 31. My parents trust me and allow me to do some things alone. | Father | 1 | 2 | 3 | 4 |
|  | Mother |  |  |  |  |
| 32. I think my parents respect my point of view. | Father | 1 | 2 | 3 | 4 |
|  | Mother |  |  |  |  |
| 33. I think my parents are willing to be with me. | Father | 1 | 2 | 3 | 4 |
|  | Mother |  |  |  |  |
| 34. I think my parents are very stingy to me, very mean. | Father | 1 | 2 | 3 | 4 |
|  | Mother |  |  |  |  |
| 35. My parents always say to me something like "If you do this I will be very sad". | Father | 1 | 2 | 3 | 4 |
|  | Mother |  |  |  |  |
| 36. My parents asked me to come home and explain to them what I was doing. | Father | 1 | 2 | 3 | 4 |
|  | Mother |  |  |  |  |
| 37. I think my parents are trying to make my youth more meaningful and colorful (such as buying me a lot of books, arranging me go to summer camp or join a club). | Father | 1 | 2 | 3 | 4 |
|  | Mother |  |  |  |  |
| 38. Parents often express to me such words as "This is the reward we get for your hard work all day?" | Father | 1 | 2 | 3 | 4 |
|  | Mother |  |  |  |  |
| 39. Parents often can not spoil me as an excuse not to meet my requirements. | Father | 1 | 2 | 3 | 4 |
|  | Mother |  |  |  |  |
| 40. If I do not do what my parents expect, it will make me uneasy on my conscience. | Father | 1 | 2 | 3 | 4 |
|  | Mother |  |  |  |  |
| 41. I think my parents have higher requirements for my academic performance, sports activities or similar things. | Father | 1 | 2 | 3 | 4 |
|  | Mother |  |  |  |  |
| 42. I can get comfort from my parents when I feel sad. | Father | 1 | 2 | 3 | 4 |
|  | Mother |  |  |  |  |
| 43. My parents punished me for no reason. | Father | 1 | 2 | 3 | 4 |
|  | Mother |  |  |  |  |
| 44. My parents allow me to do some of the things my friends do. | Father | 1 | 2 | 3 | 4 |
|  | Mother |  |  |  |  |
| 45. My parents often say to me that they don't like my performance at home. | Father | 1 | 2 | 3 | 4 |
|  | Mother |  |  |  |  |
| 46. Whenever I eat, my parents advise me or force me to eat more. | Father | 1 | 2 | 3 | 4 |
|  | Mother |  |  |  |  |
| 47. Parents often criticize me in front of others both lazy and useless. | Father | 1 | 2 | 3 | 4 |
|  | Mother |  |  |  |  |
| 48. Parents often pay attention to what kind of friends I associate. | Father | 1 | 2 | 3 | 4 |
|  | Mother |  |  |  |  |
| 49. If something happens, I am often the only one to blame among my brothers and sisters. | Father | 1 | 2 | 3 | 4 |
|  | Mother |  |  |  |  |
| 50. Parents can let me naturally develop. | Father | 1 | 2 | 3 | 4 |
|  | Mother |  |  |  |  |
| 51. Parents are often rude to me. | Father | 1 | 2 | 3 | 4 |
|  | Mother |  |  |  |  |
| 52. Sometimes even for a little trifles, parents will severely punish me. | Father | 1 | 2 | 3 | 4 |
|  | Mother |  |  |  |  |
| 53. My parents have beaten me for no reason. | Father | 1 | 2 | 3 | 4 |
|  | Mother |  |  |  |  |
| 54. My parents usually participate in my hobby activities. | Father | 1 | 2 | 3 | 4 |
|  | Mother |  |  |  |  |
| 55. I often get beat by my parents. | Father | 1 | 2 | 3 | 4 |
|  | Mother |  |  |  |  |
| 56. My parents often allow me to go where I like to go, and they don't worry too much. | Father | 1 | 2 | 3 | 4 |
|  | Mother |  |  |  |  |
| 57. My parents have strict restrictions on what I should do and what I should not do and never give in. | Father | 1 | 2 | 3 | 4 |
|  | Mother |  |  |  |  |
| 58. Parents often treat me in a way that makes me very embarrassed. | Father | 1 | 2 | 3 | 4 |
|  | Mother |  |  |  |  |
| 59. I think my parents' concern about my possible accident is exaggerated and excessive. | Father | 1 | 2 | 3 | 4 |
|  | Mother |  |  |  |  |
| 60. I feel a warm, caring and affectionate relationship with my parents. | Father | 1 | 2 | 3 | 4 |
|  | Mother |  |  |  |  |
| 61. My parents can tolerate that I have different opinions from them. | Father | 1 | 2 | 3 | 4 |
|  | Mother |  |  |  |  |
| 62. My parents often throw tantrums at me for reasons I don't know. | Father | 1 | 2 | 3 | 4 |
|  | Mother |  |  |  |  |
| 63. When I succeed in what I do, I think my parents are very proud of me. | Father | 1 | 2 | 3 | 4 |
|  | Mother |  |  |  |  |
| 64. Compared with my brothers and sisters, my parents often prefer me. | Father | 1 | 2 | 3 | 4 |
|  | Mother |  |  |  |  |
| 65. Sometimes even if the fault is mine, the parents also blame the brothers and sisters. | Father | 1 | 2 | 3 | 4 |
|  | Mother |  |  |  |  |
| 66. Parents often hug me. | Father | 1 | 2 | 3 | 4 |
|  | Mother | 1 | 2 | 3 | 4 |
